# Analysis of Magneto-Mechanical Response for Magnetization-Graded Ferromagnetic Material in Magnetoelectric Laminate

**DOI:** 10.3390/ma13122812

**Published:** 2020-06-22

**Authors:** Hai Zhou, Feihu Yu, Xueling Jiang, Caijiang Lu, Zhongqing Cao, Xiang Chen, Hongli Gao, Aichao Yang

**Affiliations:** 1 Department of Electromechanical Measuring and Controlling, School of Mechanical Engineering, Southwest Jiaotong University, Chengdu 610031, China; zhouhai131@163.com (H.Z.); yfhdmail@163.com (F.Y.); 18384278391@163.com (X.J.); zqcao@swjtu.edu.cn (Z.C.); chenxiang_189@163.com (X.C.); hongli_gao@swjtu.edu.cn (H.G.); 2 Jiangxi Electric Power Research Institute, Nanchang 330096, China; dkyyac2015@163.com

**Keywords:** piezomagnetic coefficient, magnetization-graded ferromagnetic material, magnetoelectric device

## Abstract

This paper analyzes the dynamic magneto-mechanical response in magnetization-graded ferromagnetic materials (MGFM) comprised of high-permeability Finemet and traditional magnetostrictive materials. The theoretical modeling of the piezomagnetic coefficient that depends on the bias magnetic field of MGFM is proposed by using the nonlinear constitutive model of a piezomagnetic material, the magnetoelectric equivalent circuit method, and the simulation software Ansoft. The theoretical variation of piezomagnetic coefficients of MGFM on the bias magnetic field is in good agreement with the experiment. Using the piezomagnetic coefficient in the magnetoelectric voltage model, the theoretical longitudinal resonant magnetoelectric voltage coefficients have also been calculated, which are consistent with the experimental values. This theoretical analysis is beneficial to comprehensively understand the self-biased piezomagnetic response of MGFM, and to design magnetoelectric devices with MGFM.

## 1. Introduction

Magnetoelectric (ME) laminate composites comprising of piezomagnetic and piezoelectric phases have been extensively investigated in recent years for use in potential smart devices due to their low cost, lightweight, and flexible features [1,2,3]. The principal of ME response can be determined from the product properties of the piezomagnetic and piezoelectric phases. The ME coefficient is expressed as

(1) $$\alpha_{ME}=k_{c}\times d_{m}\times d_{p}$$

where *k_c_* is a coupling factor (0 ≤ *k_c_* ≤ 1) between the two phases, *d_m_* is the piezomagnetic coefficient, and *d_p_* is the piezoelectric coefficient. The *d_m_* and *d_p_* are decided by the choice of piezomagnetic materials (Terfenol-D, Metglas, Gafenol, etc.) and piezoelectric materials (Pb(Zr_1−x_Ti_x_)O_3_ ceramics, PMN-PT single crystal, AlN films, etc.), respectively [1,2,3]. The coupling factor *k_c_*, according to previous reports, is mainly influenced by configuration [4,5,6], boundary conditions [7], and composite mode (epoxy bonding [8], magnetron sputtering [9], and spin-wave interactions [10,11,12]). The configuration and boundary conditions determine the mechanical structure of ME composites. The composite mode determines the interface coupling between two phases.

For most magnetostrictive materials with a small magnetic hysteresis, the value of *d_m_* is near zero without an external magnetic field [1,2,3,4,5,6,7,8,9]. Thus, a magnetoelectric composite requires a DC biased magnetic field (*H*_dc_) to obtain an enhanced value of *d_m_* [13]. Generally, the *H*_dc_ is provided by a pair of permanent magnets resulting in a possible noise source in the sensor array, a reduction of the spatial resolution, and an increase in the device volume.

For weakening the dependence of *H*_dc_, the self-biased piezomagnetic effect has been presented by researchers. Using the inherent remanence of magnetic material is an effective way [14,15,16], but the self-biased ME coupling is still weak. The antiferromagnetic–ferromagnetic exchange coupling effect is the other effective method [17,18], but the processing technology is complex. Beyond that, the ME laminate with magnetization-graded ferromagnetic materials (MGFM) consisting of high-permeability ribbons and traditional magnetostrictive materials also has an effective self-biased ME response [19,20,21,22,23]. Up until now, the experimental self-biased ME response for the asymmetric piezoelectric/MGFM laminate and the symmetric MGFM/piezoelectric/MGFM structure have been studied [19,20,21,22,23]. However, up until now, few studies have focused on the theoretical magneto-mechanical response for MGFM.

In this paper, we analyze the magneto-mechanical response of MGFM consisting of Finemet and traditional piezomagnetic layers (Ni or FeNi alloy). The theoretical modeling of the magneto-mechanical behavior of MGFM is established using the nonlinear piezomagnetic constitutive model and the method of the equivalent circuit. Compared with the experimental data, the bias field dependences of piezomagnetic coefficients are in good agreement with the calculation from the presented model.

## 2. Theoretical Analysis

### 2.1. Dynamic Effective Piezomagnetic Coefficient d_33,m_ of MGFM

**Figure 1:**
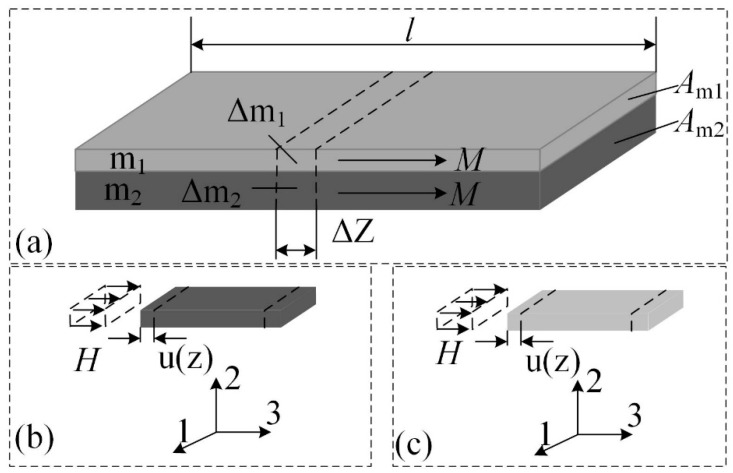
(**a**) Schematic diagram of magnetization-graded ferromagnetic material FeCuNbSiB/Ni. The local coordinate systems in (**b**) FeCuNbSiB ribbon and (**c**) Ni plate when the magnetic field is applied along the longitudinal 3-direction. The symbol *M* is magnetization. The *m*_1_ and *m*_2_ is the mass of the FeCuNbSiB ribbon and Ni plate, respectively. The Δ*m*_1_ and Δ*m*_2_ are small mass units in the FeCuNbSiB ribbon and Ni plate, respectively. The *A_m_*_1_ and *A_m_*_2_ is the cross-sectional area of the FeCuNbSiB ribbon and Ni plate, respectively. The *l* is the length of FeCuNbSiB/Ni. The *H* is the applied magnetic field. The *u*(*z*) is the displacement.

The MGFM FeCuNbSiB/Ni is shown in Figure 1. The phase *m*_1_ is Finemet (FeCuNbSiB), phase *m*_2_ is traditional magnetostrictive material (Ni or FeNi). According to the principle of wave mechanics, the displacement direction of the laminate is the same as the propagation direction of the vibration, so the wave formed by elastic vibration is a longitudinal wave. Under the free boundary conditions, the MGFM vibrates freely.

Then, the mechanical vibration equation can be derived as follows [24,25]

(2) $$\left\{ \begin{array}{l} F_{1}=Z_{1}{\overset{•}{u}}_{1}+Z_{2}({\overset{•}{u}}_{1}-{\overset{•}{u}}_{2})+(\varphi_{m1}+\varphi_{m2})H_{3}\\ F_{2}=-Z_{1}{\overset{•}{u}}_{2}+Z_{2}({\overset{•}{u}}_{1}-{\overset{•}{u}}_{2})+(\varphi_{m1}+\varphi_{m2})H_{3} \end{array}\right.$$

where $Z_{1}=j\overset{-}{\rho }\nu A\tan\frac{kl}{2}$, $Z_{2}=\frac{\overset{-}{\rho }\nu A}{j\sin kl}$, $\varphi_{m1}=\frac{A_{m1}d_{33,m1}}{s_{33,m1}^{H}}$, $\varphi_{m2}=\frac{A_{m2}d_{33,m2}}{s_{33,m2}^{H}}$, *F*_l_ and *F*_2_ are forces at the two end faces of the structure, and the ${\dot{u}}_{1}$ and ${\dot{u}}_{2}$ are the corresponding displacements. $\bar{\rho }=\left(\rho_{m1}t_{m1}+\rho_{m2}t_{m2}\right)/t$ is the average density of the laminate. $\varphi_{m1}$ and $\varphi_{m2}$ are the magneto-elastic coupling factor. The *d*_33__,*m*1_ and *d*_33__,*m*__2_ is the longitudinal piezomagnetic coefficient of FeCuNbSiB and Ni, respectively. $\bar{\rho }$ is the average density of the laminate. The *k* is the wavenumber and *v* is the sound velocity. $s_{33,m1}^{H}$ and $s_{33,m2}^{H}$ is the elastic compliance under constant *H* for FeCuNbSiB and Ni, respectively.

**Figure 2:**
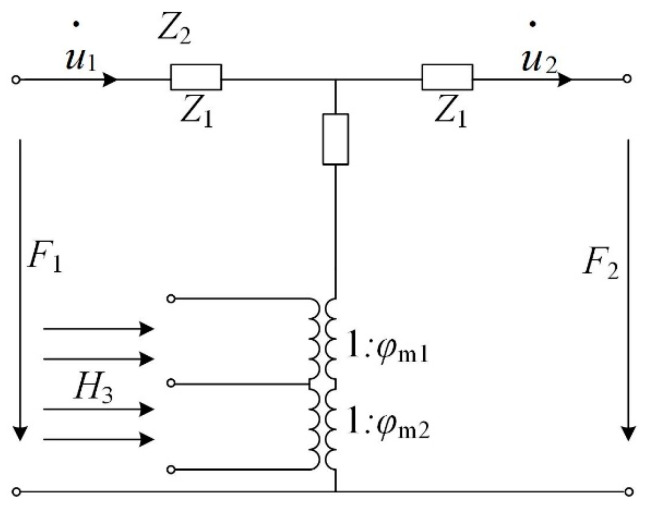
Magneto-elastic equivalent circuit of the magnetization-graded ferromagnetic materials (MGFM).

In this paper, the method of equivalent circuit is used to deal with the magneto-elastic interaction, as shown in Figure 2.

From the equivalent circuit, the relationship for *φ_m_* of FeCuNbSiB/Ni, *φ_m_*_1_ of FeCuNbSiB phase and *φ_m_*_2_ of Ni phase is

(3) $$\varphi_{m}=\varphi_{m1}+\varphi_{m2}$$

Then

(4) $$\frac{(A_{m1}+A_{m2})d_{33,m}}{\bar{s}}=\frac{A_{m1}d_{33,m1}}{s_{33,m1}^{H}}+\frac{A_{m2}d_{33,m2}}{s_{33,m2}^{H}}$$

where $\bar{s}$ is the average compliant coefficient and $\frac{1}{\bar{s}}=\frac{t_{m1}}{(t_{m1}+t_{m2})s_{33,m1}^{H}}+\frac{t_{m2}}{(t_{m1}+t_{m2})s_{33,m2}^{H}}$. *t_m_*_1_ and *t_m_*_2_ are the thicknesses of FeCuNbSiB and Ni, respectively. So, the *d*_33,*m*_ of MGFM is

(5) $$d_{33,m}=\frac{t_{m1}d_{33,m1}s_{33,m2}^{H}+t_{m2}d_{33,m2}s_{33,m1}^{H}}{t_{m1}s_{33,m2}^{H}+t_{m2}s_{33,m1}^{H}}$$

### 2.2. Effective Dynamic Piezomagnetic Coefficient d_33,m__1_ of FeCuNbSiB in MGFM

According to the nonlinear magnetostrictive constitutive relationship, the *d*_33,*m*_ of magnetostrictive material is [26]

(6) $$d_{33,m}=\frac{\partial \varepsilon }{\partial H}=\frac{\partial \varepsilon }{\partial M}\frac{\partial M}{\partial H}=2\lambda_{s}(\coth(\eta H)-\frac{1}{\eta H})\times [\eta (1-\coth{\left(\eta H\right)}^{2})+\frac{1}{\eta H^{2}}]$$

where *M* is the magnetization, *ε* is the stain, *λ_s_* is the saturation magnetostrictive coefficient and *H* is the external magnetic field. *η =* 3*χ_m_*/*M_s_ χ_m_* is the initial magnetic susceptibility, *M_s_* is the saturation magnetization.

Due to the remanent magnetism of Ni, an additional magnetic field *H_f_* in FeCuNbSiB is generated. Thus, the effective magnetic field of the FeCuNbSiB ribbons in the FeCuNbSiB/Ni laminate (*H_eff,m_*_1_) is

(7) $$H_{eff,m1}=\frac{H_{\mathrm{dc}}+H_{\mathrm{ac}}+H_{f}}{1+N_{d,m1}(\mu_{r,m1}-1)}$$

where *N*_d,*m*1_ is the demagnetizing factor of FeCuNbSiB. *H*_dc_ is the bias magnetic field. *H*_ac_ is the alternating current magnetic field. Therefore, the effective dynamic effective piezomagnetic coefficient *d*_33,*m*1_ of FeCuNbSiB in the FeCuNbSiB/Ni laminate is

(8) $$d_{33,m1}=2\lambda_{s,m1}(\coth(\eta_{m1}H_{eff,m1})-\frac{1}{\eta_{m1}H_{eff,m1}})\times [\eta_{m1}(1-\coth{\left(\eta_{m1}H_{eff,m1}\right)}^{2})+\frac{1}{\eta_{m1}{\left(H_{eff,m1}\right)}^{2}}]$$

In order to obtain the value of *H_f_*, we use the magnetic field simulation software, Ansoft 11.0. In the simulation, the residual magnetization of Ni is assumed as the parameter input, so Ni provides the magnetic field *H_f_*. For magnetostrictive Ni with cubic magnetocrystalline anisotropy and $\left|K_{1}\right|<M_{s}^{2}$, the remanent magnetization is *M_r_* = 0.866*M_s_*, *μ*_0_*M_s_* = 0.616 T. The simulation settings are as follows; Ni: *μ*_0_*M_r_* = 0.534 T, *μ_r_* = 220; dimensions are 12 × 6 × 1 mm^3^; FeCuNbSiB: *μ_r_* = 30,000; dimensions are 12 × 6 × 0.120 mm^3^.

**Figure 3:**
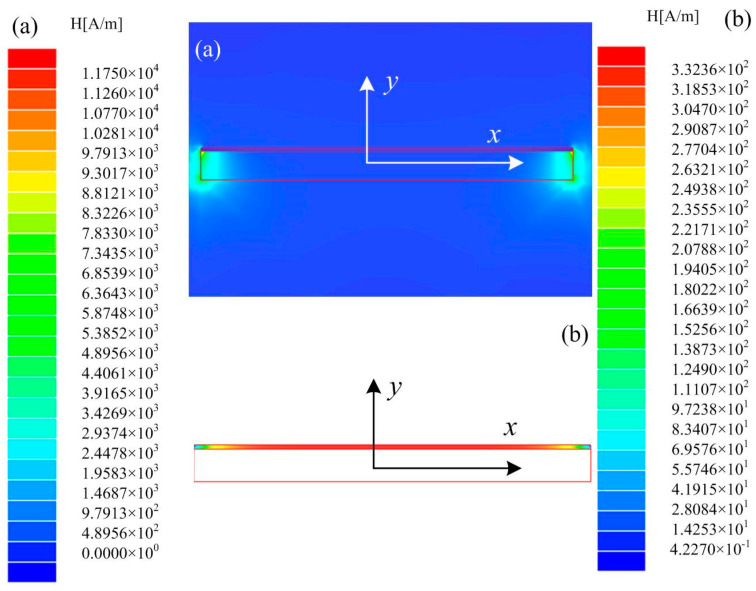
(**a**) The distribution diagrams of magnetic fields of the FeCuNbSiB/Ni laminate, and (**b**) The distribution diagrams of magnetic fields of the FeCuNbSiB ribbon in the FeCuNbSiB/Ni laminate.

Figure 3a,b illustrates the distributions of magnetic fields of the FeCuNbSiB/Ni laminate and the FeCuNbSiB ribbon in FeCuNbSiB/Ni, respectively. In the absence of *H*_dc_, it can be clearly displayed from Figure 3 that the Ni plate provided a *H*_dc_ to FeCuNbSiB due to the residual magnetization. When the layer of FeCuNbSiB ribbon *L* = 1, *H_f_* = 276.78 (A/m).

### 2.3. Dynamic Effective Piezomagnetic Coefficient d_33,m__2_ of Ni Plate in MGFM

For FeCuNbSiB as a soft magnetic material, the permeability *μ_rf_* = 30,000, saturation magnetization *μ*_0_*M_s_* = 1.45 T, and the anisotropic constant is −30,000 J/m^3^. Comparatively, for pure Ni, the permeability (*μ_r_* = 60) and saturation magnetization (*μ*_0_*M_s_* = 0.616 T) are lower, and the anisotropic constant (100 J/m^3^) is smaller. The magnetic properties of the FeCuNbSiB ribbons and the Ni plate are significantly different, which results in an additional magnetic field being generated in the Ni plate due to the flux concentration effect of the high-permeability FeCuNbSiB ribbons [9,23]. According to previous reports [9,23], a magnetic material with high-permeability can be assumed as a static magnetic source, which results in the flux concentration effect and generates an effective magnetic field around it. Thus, the effective magnetic field in Ni of FeCuNbSiB/Ni (*H_eff,m_*_2*f*_) is larger than that of a single Ni plate without FeCuNbSiB (*H_eff,m_*_2*n*_) due to the flux concentration effect of FeCuNbSiB. Here, we assume that the coefficient of the flux concentration effect is *δ*. So, both *H*_dc_ and *H*_ac_ in Ni are amplified *δ* times. In general, *H*_dc_ is far larger than the *H*_ac_. Furthermore, the internal magnetic field in the Ni plate is influenced by the demagnetizing field *H_d_* which is completely opposite to the magnetization. The effective magnetic field *H*_eff,*m*2*n*_ of the single Ni plate and *H*_eff,*m*2*f*_ of Ni in the FeCuNbSiB/Ni laminate are

(9a) $$H_{eff,m2n}=\frac{H_{\mathrm{dc}}+H_{\mathrm{ac}}}{1+N_{d,m2}(\mu_{r,m2}-1)}$$

(9b) $$H_{eff,m2f}=\frac{\delta (H_{\mathrm{dc}}+H_{\mathrm{ac}})}{1+N_{d,m2}(\mu_{r,m2}-1)}$$

From Equation (9a) and (9b), the piezomagnetic coefficient of single Ni *d*_33,*m*2*n*_*,* and Ni in FeCuNbSiB/Ni *d*_33,_*_m_*_2*f*_ are

(10a) $$d_{33eff,m2n}=2\lambda_{s,m2}(\coth(\eta_{{}_{m2}}H_{eff,m2n})-\frac{1}{\eta_{{}_{m2}}H_{eff,m2n}})\times [\eta_{m2}(1-\coth{\left(\eta_{m2}H_{eff,m2n}\right)}^{2})+\frac{1}{\eta_{{}_{m2}}{\left(H_{eff,m2n}\right)}^{2}}]$$

(10b) $$d_{33eff,m2f}=2\delta \lambda_{s,mf}(\coth(\eta_{{}_{m2}}H_{eff,m2f})-\frac{1}{\eta_{{}_{m2}}H_{eff,m2f}})\times [\eta_{m2}(1-\coth{\left(\eta_{m2}H_{eff,m2f}\right)}^{2})+\frac{1}{\eta_{{}_{m2}}{\left(H_{eff,m2f}\right)}^{2}}]$$

**Figure 4:**
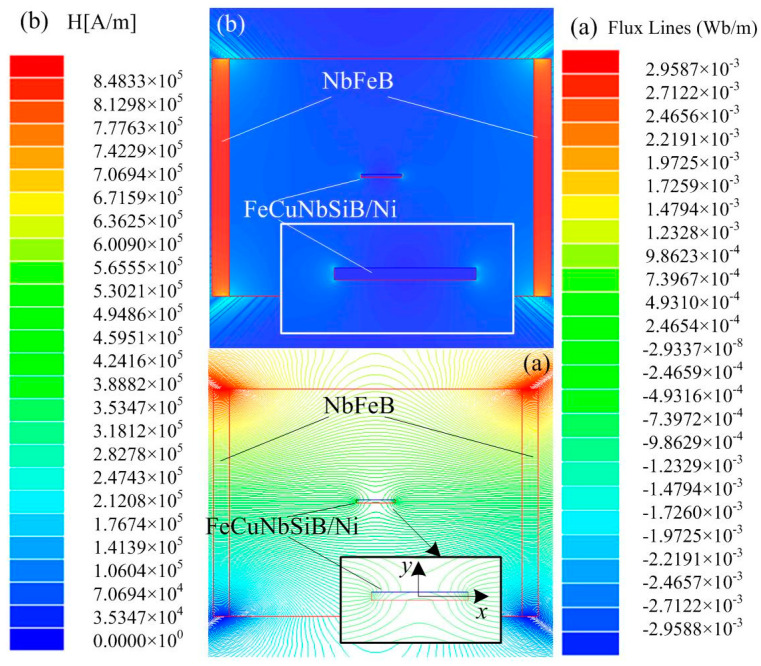
When the layers of the FeCuNbSiB ribbon *L* = 1, the distribution diagrams of (**a**) flux lines and (**b**) magnetic field of the FeCuNbSiB/Ni laminate.

According to the principle of magnetic charge, the magnetic charges of the Ni plate are gathered and distributed at the two end faces. So the amplitude of the magnetic field at the face ends of the Ni plate can reflect its magnetization state, assuming that the amplitude of the magnetic field at the end of the single Ni plate is *H*_Ni_ and Ni in FeCuNbSiB/Ni is *H*_F-Ni_. In order to obtain the values of *H*_Ni_ and *H*_F-Ni_, we use the Ansoft 11.0 software package (2D magnetostatic simulation) so we can obtain *δ* = *H*_F-Ni_/*H*_N__i_. The two-dimensional simulation parameters are set as follows: permanent magnet NdFeB-N35 (75 mm length × 5 mm width, the distance of two magnets is 90 mm); Ni (*μ* = 220, 12 mm length × 1 mm height); and FeCuNbSiB (*μ* = 3 × 10_4_, 75 mm length × 0.03 mm height). When the layers of FeCuNbSiB ribbon *L* = 1, the distribution of flux lines of FeCuNbSiB/Ni; the distribution of the magnetic field of the FeCuNbSiB/Ni laminate are shown in Figure 4.

**Figure 5:**
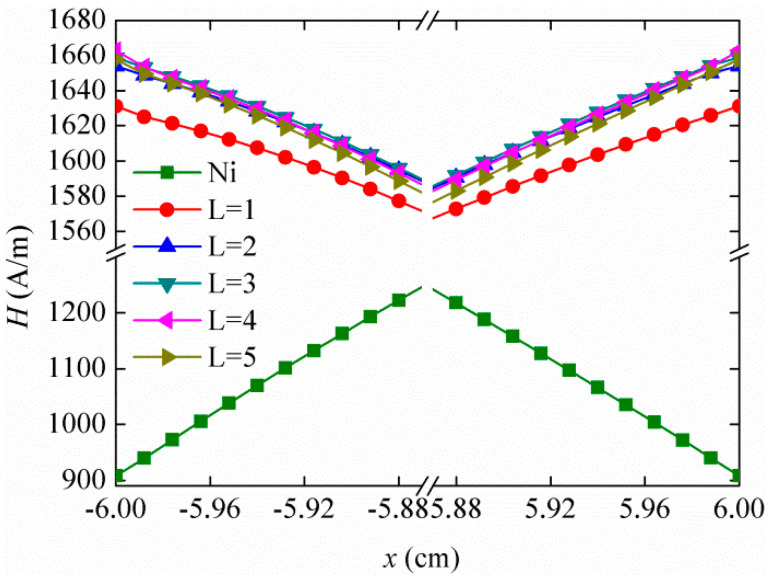
The amplitude of the magnetic field along *x*-direction for the single Ni plate and Ni in FeCuNbSiB/Ni (*L* = 1–5).

As shown in Figure 5, when centerline *x* changes, the magnetic field *H* curves along the *x*-direction near the two ends of the Ni plate. Clearly, the magnetic field of the Ni plate in FeCuNbSiB/Ni is far greater than that in the single Ni plate, which is attributed to the flux concentration effect of FeCuNbSiB. For a single Ni plate, *H*_Ni_ is 908 (A/m) at the end face. When the FeCuNbSiB layer number *L* = 1–5, *H*_F-Ni_ at the end faces of the Ni plate in FeCuNbSiB/Ni are 1631.15395 (A/m), 1653.96385 (A/m), 1658.66092 (A/m), 1662.78705 (A/m), and 1657.69218 (A/m), respectively. When *L* = 4, the end face magnetic field intensity is the maximum. Therefore, according to the simulation results, the magnetic field convergence factor is *δ* = *H*_F-Ni_/*H*_Ni_, which is about 1.83 times.

## 3. Results and Discussion

**Figure 6:**
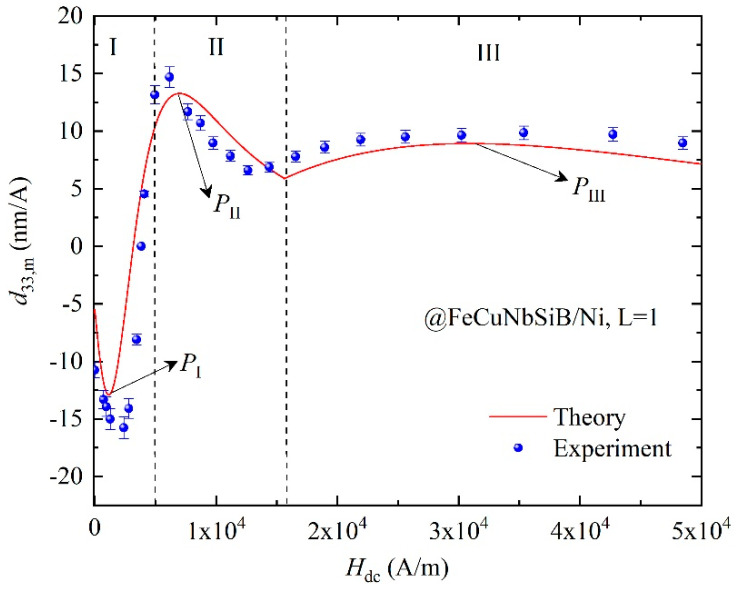
The calculated and experimental *d*_33*,m*_ as a function of *H*_dc_ for the FeCuNbSiB/Ni laminate. The layer of FeCuNbSiB is one (*L* = 1).

In the present work, we compare the calculated results to the experiments to prove the effectiveness of our model for the magneto-mechanical and ME coupling characteristics. Substituting the material’s parameters shown in Table 1 into Equations (5), (8), (10a) and (10b), the theoretical results of *d*_33,*m*_ versus *H*_dc_ for FeCuNbSiB/Ni can be obtained, as shown in Figure 6. The FeCuNbSiB is a positive magnetostrictive material, and Ni is a negative magnetostrictive material. In the calculation, *d*_33,*m*1_ and *d*_33,*m*2_ in Equation (5) are converse.

In experiments, the Doppler vibrometer (Polytec OFV-5000) was used to measure the vibration velocity *v* at the end faces of the FeCuNbSiB/Ni laminate, then the *d*_33,*m*_ was calculated by *d*_33,*m*_ = *dλ*/*dH* = *v*/(*πƒlH*_ac_). The *H*_ac_ is the external AC magnetic field, ƒ is the vibration frequency, and *l* is the length. It shows clearly that the calculated *d*_33,*m*_ agrees well with the experimental data, and a large zero-biased *d*_33,*m*_ of FeCuNbSiB/Ni can be achieved. The errors in experimental data originally from the magnetization state of piezomagnetic material is inconsistent in each measurement during all the processes of repeated measurements.

From Figure 6, there is an error between the calculation result and the experimental values. The reason for this phenomenon is that the experimental device was not fully demagnetized in the actual experimental process, and some simulation parameters are used in the theoretical calculation. We can obtain the results:

(i) For the outstanding soft magnetic performance of FeCuNbSiB, the magneto-mechanical coupling of FeCuNbSiB is larger than Ni in Section 1 (*d*_33,*m*1_ > *d*_33,*m*2_). As *H*_dc_ increases, the *d*_33,*m*1_ increases rapidly, and the maximum value *P*_I_ = 12.9 nm/A is obtained under the optimal *H*_dc_ = 1184 A/m.
(ii) When *H*_dc_ increases to a certain value, the magneto-mechanical effects of FeCuNbSiB and Ni cancel each other out, and the dynamic *d*_33,*m*_ appears at the zero-cross point as shown in Figure 6.
(iii) Due to the flux concentrate effect of FeCuNbSiB, the internal magnetic moments of Ni rotate rapidly. Then, the *d*_33,*m*2_ becomes larger. In Section 2 of Figure 6, the *d*_33,*m*_ is originated from the magneto-mechanical coupling of Ni with the flux concentrate effect. After the zero-crossing point, the *d*_33,*m*_ rapidly increases to the maximum value *P*_II_. However, when *H*_dc_ increases to a certain value, FeCuNbSiB gradually saturates, the flux concentration effect is abate, which results in the appearance of the downward trend in the II area.
(iv) With the increase of *H*_dc_, the flux concentration effect gradually decreases, so in Section 3 of Figure 6, the *d*_33,*m*_ is attributed to the magneto-mechanical coupling of Ni itself. From Equation (10a) and (10b), we assume that *d*_33,*m*2*f*_ = *d*_33,*m*2*n*_ when *H*_dc_ = *H_n_,* and*d*_33,*m*2*f*_ is far larger than *d*_33,*m*2*n*_ when *H*_dc_ < *H_n_*. As *H*_dc_ increases gradually, the flux concentration effect disappears when *H*_dc_ > *H_n_*. So for the *d*_33,*m*_ of the Ni plate in the theoretical calculation, *d*_33,m2_ =*d*_33,*m*2*f*_ when *H*_dc_ < *H_n_* and *d*_33,m2_ = *d*_33,*m*2*n*_ when *H*_dc_ > *H_n_*. From Figure 6, the magnetic field *H_n_* for *d*_33,*m*2*f*_ = *d*_33,*m*2*n*_ in Equation (10a) and (10b) is ~12,800 (A/m) in experiments, and is ~15,600 (A/m) in theory.

Using the method of the magneto-mechanical-electric equivalent circuit, the longitudinal resonant ME voltage coefficient *α_ME,_*_r_ for L–T mode laminate structure is given by [24,25]

(11) $$\alpha_{ME,r}=\left|\frac{dV}{tdH_{3}}\right|=\frac{8Q_{m}}{\pi^{2}}\frac{n(1-n)d_{33,m}d_{31,p}}{\varepsilon_{33}[f(1-k_{31}^{2})s_{11}^{E}+(1-n)s_{33}^{H}]}$$

where *Q_m_* is the mechanical quality factor, *n* is the volume ratio of the magnetostrictive phase, *ε*_33_ is the permittivity tensor, *d*_33,*m*_ is the piezomagnetic coefficient, *d*_31,*p*_ is the piezoelectric coefficient, *k*_31_ is the electromechanical coupling coefficient of the piezoelectric material, $s_{11}^{E}$ is the elastic compliance of the piezoelectric phase, and $s_{33}^{H}$ is the elastic compliance of the piezomagnetic phase.

**Figure 7:**
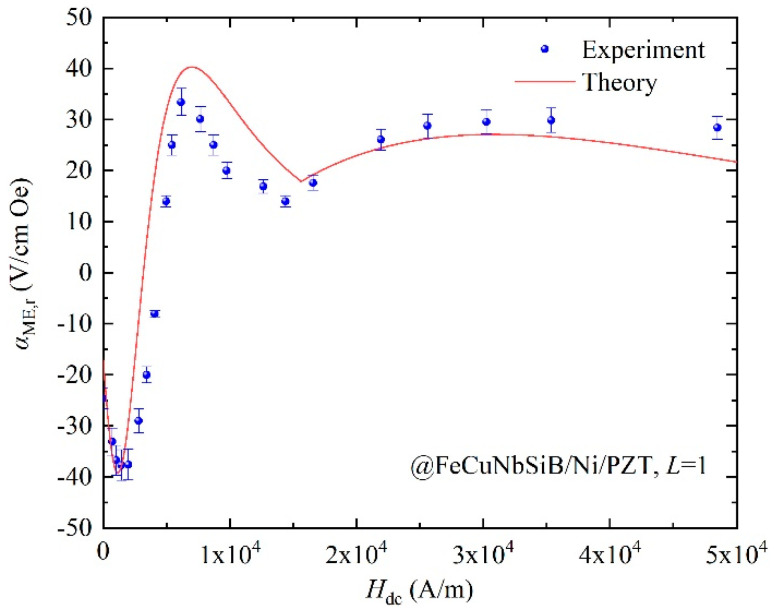
The theoretical and experimental *α_ME,_*_r_ vs. *H*_dc_ for the FeCuNbSiB/Ni/PZT composite.

Using Pb(Zr_1−x_Ti_x_)O_3_ (PZT) ceramic as the piezoelectric material, the ME composite FeCuNbSiB/Ni/PZT can be obtained. Taking the material’s parameters in Table 1 and Equation (5) into Equation (11), the theoretical *α_ME,_*_r_ can be obtained, as shown in Figure 7. For comparison, the experimental data for FeCuNbSiB/Ni/PZT with *L* = 1 are used, as shown in Figure 7. It can be seen from the figure that the theoretical calculation and experimental value of *α_ME,_*_r_ is consistent with the variation trend of the *H*_dc_. From Equation (11), one can derive *α_ME,_*_r_ ∝ *d*_33,*m*_. Therefore, the curve of *α_ME,_*_r_ vs. *H*_dc_ in Figure 7 is consistent with that of *d*_33,*m*_ vs. *H*_dc_ in Figure 6. The theoretical calculation result is different from the experimental result. The reason is that the experimental device is not demagnetized in the actual experimental process. In addition, the composite structure of FeCuNbSiB/Ni/PZT has a certain thickness of glue, which was not considered in the theoretical calculation results.

In previous work, FeCuNbSiB shows positive magnetostriction and Ni shows negative magnetostriction. In order to check the validity of the theoretical model, the other magnetostrictive material FeNi with positive magnetostrictive properties was chosen to replace Ni. Then, we also compared the theoretical results with the experimental data using MGFM FeCuNbSiB/FeNi and ME laminate FeCuNbSiB/FeNi/PZT.

**Figure 8:**
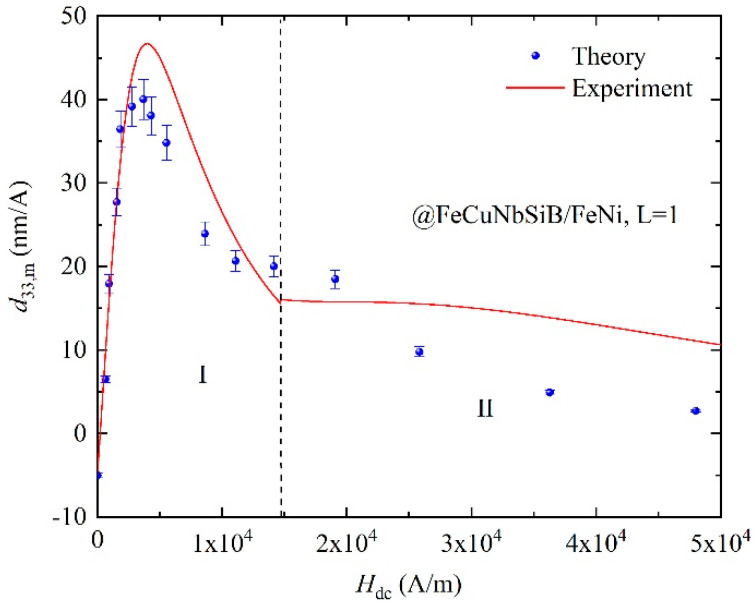
Dynamic piezomagnetic coefficient *d*_33*,m*_ of FeCuNbSiB/FeNi as a function of *H*_dc_. The layer of FeCuNbSiB is one (*L* = 1).

Substituting the material’s parameters, shown in Table 1, into Equations (5), (8), (10a) and (10b), the calculated *d*_33,*m*_ versus *H*_dc_ for FeCuNbSiB/FeNi is achieved, as shown in Figure 8. The result in Figure 8 is different from that of Figure 6. The reason is that the Ni shows negative magnetostriction and FeNi shows positive magnetostriction. In Section 1, the *d*_33,m_ of FeCuNbSiB/FeNi originates from *d*_33,*m*1_ of FeCuNbSiB and *d*_33,*mf*_ (Equation (10b)) of FeNi with the flux concentration effect. In Section 2, the *d*_33,*m*_ of FeCuNbSiB/FeNi originates from *d*_33,*mn*_ (Equation (10a)) of FeNi.

**Figure 9:**
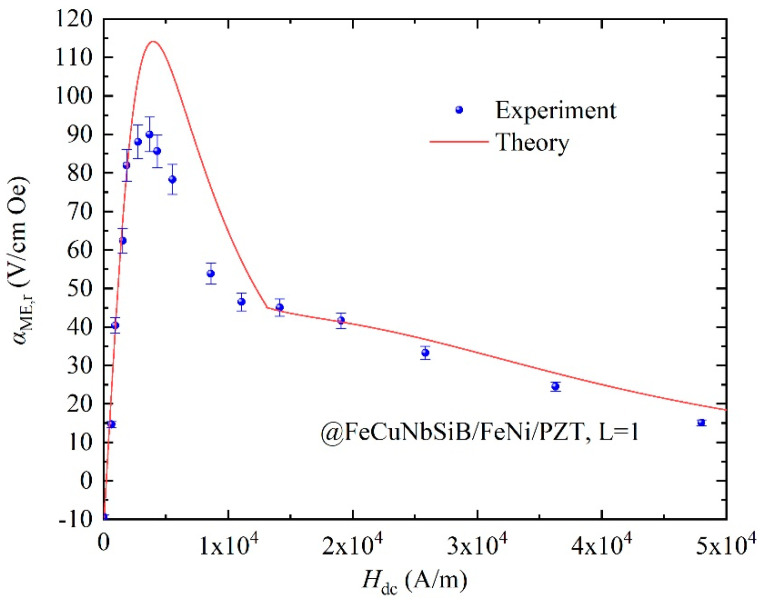
The theoretical and experimental *α_ME_*_,__r_ as a function of *H*_dc_ for FeCuNbSiB/FeNi/PZT.

To make further efforts to verify the proposed theoretical model, the FeCuNbSiB/FeNi/PZT composite is used, and the calculated and experimental data are shown in Figure 9. The curve of *α_ME,_*_r_ vs. *H*_dc_ in Figure 9 is consistent with that of *d*_33,*m*_ vs. *H*_dc_ in Figure 8. The theoretical result for *d*_33,*m*_, and *α_ME,_*_r_ agrees well with those of experiments. Clearly, the proposed theoretical models are also proven to be useful for the other MGFM and ME laminated composite with MGFM.

## 4. Conclusions

According to the nonlinear constitutive model of piezomagnetic material, magnetoelectric equivalent circuit method, and simulation software Ansoft, a theoretical model about *d*_33,*m*_ vs. *H*_dc_ is effectively and accurately established for MGFM. The theoretical *d*_33,*m*_ of FeCuNbSiB/Ni, or FeCuNbSiB/FeNi has been compared with the experimental data. By using the presented piezomagnetic model of MGFM, the resonant ME voltage coefficient *α_ME,_*_r_ as a function of *H*_dc_ for FeCuNbSiB/Ni/PZT or FeCuNbSiB/FeNi/PZT composite has been investigated. The theoretical calculated result agrees well with that of the experiments. Therefore, the proposed piezomagnetic model will provide an effective tool to fully understand the dynamic magneto-mechanical behavior of MGFM and self-biased ME coupling of composites with MGFM. It is useful to guide the designing of ME devices with MGFM, such as magnetic sensors and energy harvesters.

## Figures and Tables

**Table 1:** Material parameters of the FeCuNbSiB ribbon, Ni plate, FeNi plate, and the Pb(Zr_1−x_Ti_x_)O_3_ (PZT) plate.

Material	*χ_m_*	*μ*_0_*M*_s_ (T)	*λ_s_* (ppm)	*L × w × t* (mm^3^)	*s*_33_ (10^–12^ m^2^/N)	*ε*_33_/*ε*_0_	*ρ* (g/cm^3^)	*Q*_mech_
Ni	200	0.616	40	12 × 6 × 1	4.9		8.9	150
FeCuNbSiB	4 × 10^5^	1.25	2.7	12 × 6×0.03	5.2		7.25	1000
FeNi	700	0.75	20	12 × 6 × 0.6	5		8	200
PZT				12 × 6 × 1	15.3	1750	7.75	1000
